# Retrieval cues that trigger reconsolidation of associative fear memory are not necessarily an exact replica of the original learning experience

**DOI:** 10.3389/fnbeh.2015.00122

**Published:** 2015-05-18

**Authors:** Marieke Soeter, Merel Kindt

**Affiliations:** ^1^Department of Clinical Psychology, University of AmsterdamAmsterdam, Netherlands; ^2^Amsterdam Brain and Cognition, University of AmsterdamAmsterdam, Netherlands

**Keywords:** fear memory, reconsolidation, updating, learning history, abstract retrieval, anxiety disorders

## Abstract

Disrupting the process of memory reconsolidation may point to a novel therapeutic strategy for the permanent reduction of fear in patients suffering from anxiety disorders. However both in animal and human studies the retrieval cue typically involves a re-exposure to the original fear-conditioned stimulus (CS). A relevant question is whether abstract cues not directly associated with the threat event also trigger reconsolidation, given that anxiety disorders often result from vicarious or unobtrusive learning for which no explicit memory exists. Insofar as the fear memory involves a flexible representation of the original learning experience, we hypothesized that the process of memory reconsolidation may also be triggered by abstract cues. We addressed this hypothesis by using a differential human fear-conditioning procedure in two distinct fear-learning groups. We predicted that if fear learning involves discrimination on basis of perceptual cues within one semantic category (i.e., the perceptual-learning group, *n* = 15), the subsequent ambiguity of the abstract retrieval cue would not trigger memory reconsolidation. In contrast, if fear learning involves discriminating between two semantic categories (i.e., categorical-learning group, *n* = 15), an abstract retrieval cue would unequivocally reactivate the fear memory and might subsequently trigger memory reconsolidation. Here we show that memory reconsolidation may indeed be triggered by another cue than the one that was present during the original learning occasion, but this effect depends on the learning history. Evidence for fear memory reconsolidation was inferred from the fear-erasing effect of one pill of propranolol (40 mg) systemically administered upon exposure to the abstract retrieval cue. Our finding that reconsolidation of a specific fear association does not require exposure to the original retrieval cue supports the feasibility of reconsolidation-based interventions for emotional disorders.

## Introduction

Reconsolidation processes allow for the modification of a previously formed fear memory when the environment requires behavioral adaptation (e.g., Dudai, 2009; Nader and Hardt, 2009). Pavlovian fear conditioning—in which a neutral conditioned stimulus (CS) is paired with a noxious unconditioned stimulus (US) is frequently used for studying reconsolidation. A series of human conditioning studies convincingly showed that disrupting reconsolidation by a noradrenergic β-blocker neutralized the fear arousing aspects of a reactivated cue that was previously associated with an aversive event (Kindt et al., 2009; Soeter and Kindt, 2010, 2011, 2012a,b; Sevenster et al., 2012, 2013, 2014). Fear reduction was not restricted to this reactivated feared cue but instead generalized to other exemplars of the same semantic category (e.g., pictures and words—Soeter and Kindt, 2011, 2012a). Generalization of the fear-erasing effects is relevant for basic research on learning and memory as well as for the feasibility of reconsolidation-based treatments in clinical practice considering that fear generalization is a key feature of anxiety disorders (e.g., LaBar and Cabeza, 2006; Lissek et al., 2008). A related question is whether cues that were *not* present during the fear learning occasion as such—but solely refer to the CS—may also trigger reconsolidation. Both in animal and human studies on memory reconsolidation the original CS is typically utilized as retrieval cue (Kindt et al., 2009; Nader and Hardt, 2009; Sevenster et al., 2013). For anxiety disorders, however, it is not always self-evident which cues are central to the underlying fear memory, as they may also result from vicarious or unobtrusive learning for which no explicit memory exists. If disrupting reconsolidation is to be developed as clinical intervention, a relevant question is whether reconsolidation is restricted to cues that are similar to the original encoding experiences or alternatively whether more abstract representations referring to a super-ordinate category of the original feared stimulus may also trigger reconsolidation.

The function of associative fear memory can be best understood from an adaptive, future-oriented perspective. At the core of this perspective lies the idea that learning experiences are flexibly represented in memory thereby permitting the ability to manipulate stored representations to serve adaptation to a complex and ever changing environment (Dudai, 2009; Buckner, 2010). Given that a previously encountered threat can appear in many forms, the conditioned defensive behavior should also extend towards other exemplars of the original learning experience (Mineka, 1992; Dunsmoor et al., 2012). The fear-conditioning paradigm is well-suited to test the generalization of associative fear learning by assessing defensive responding to other stimuli than the original fear conditioned stimulus (CS+). Systematic tests of generalization were initially developed in animals and later translated to humans.Whilst decades of animal conditioning research have focused on the perceptual similarity and discriminability of a conditioned stimulus (CS+), it became only recently evident that fear generalization depends not solely on perceptual similarities, but also on the conceptual properties of the associative fear learning experience (Dunsmoor et al., 2009, 2012; Soeter and Kindt, 2012a; Dunsmoor and LaBar, 2013). For instance we previously showed that aversive learning experiences with a single specific picture cue results in fear generalization to another picture or word cue referring to the same semantic category as the original CS (Soeter and Kindt, 2012a). Also Dunsmoor et al. (2013) showed fear generalization to super-ordinate categories but only after learning experiences with multiple exemplars of the same semantic object category. In view of the adaptive and dynamic nature of memory, in which memory is not only flexibly represented but may also change upon retrieval, we predict that an abstract representation of the original learning experience may also trigger the process of memory reconsolidation.

Given that retrieval is not sufficient for memory destabilization (Sevenster et al., 2012, 2014; Barreiro et al., 2013), the question then arises under which conditions an abstract stimulus will trigger reconsolidation. More specifically reconsolidation may only occur when a retrieval session produces an anticipation of threat that in turn generates a mismatch between what is expected and what actually happens. Both the threat expectation and mismatch are determined by the interaction between the learning history and the available information during reactivation (Pedreira et al., 2004; Forcato et al., 2009; Sevenster et al., 2013). Hence we hypothesized that the question as to whether an abstract retrieval cue would trigger memory reconsolidation depends on the previous learning experience. Only if the fear learning experience gives rise to a generalized representation of the original fear CS, an abstract retrieval cue might potentially trigger memory reconsolidation.

**Figure 1:**
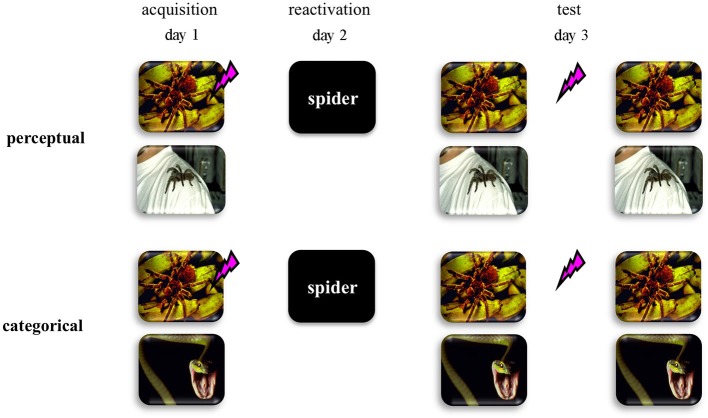
**Schematic representation of the experimental design for the perceptual discriminative-learning and categorical discriminative-learning group**. CSs are depicted as images. Unconditioned stimulus (US) is depicted as a lightning bolt. During acquisition (day 1), one of two stimuli (CS1+) was repeatedly paired with an aversive electric stimulus (US), while the other stimulus (CS2−) was not. Whereas we used two pictures of spiders as CSs in the perceptual-learning group, two stimuli of different stimulus categories served as CSs in the categorical-learning group. On day 2 the memory was reactivated through a word referring to a categorical representation of the CS1 (i.e., CS1-R). After the presentation of the CS1-R stimulus, all participants received an oral dose of 40 mg propranolol—a β-adrenergic receptor antagonist known to disrupt reconsolidation (Dębiec and LeDoux, 2004; Kindt et al., 2009; Sevenster et al., 2013).

Here we addressed this hypothesis by using a differential human fear conditioning procedure in two distinct fear-learning groups (see Figure 1). We reasoned that if fear learning involves discrimination on basis of perceptual cues within one semantic category (i.e., two pictures of spiders)—from now on referred to as perceptual-learning group—no generalized fear network will be formed. As a consequence, an abstract retrieval cue would refer to both the fear CS and the safety stimulus, and would not trigger reconsolidation of the fear memory. On the other hand, if fear learning involves the discrimination between semantic categories (i.e., picture of a spider vs. picture of a snake)—from now on referred to as categorical-learning group—a generalized fear network would be formed even on basis of multiple experiences with only one fear CS. In this case, an abstract retrieval cue would unequivocally reactivate the fear memory and may subsequently trigger memory reconsolidation. Hence we predicted that in the categorical-learning group—and not in the perceptual-learning group—reactivation by an abstract word cue of the fear CS would trigger reconsolidation of the original fear memory representation, such that propranolol administration upon retrieval could interfere with the process of memory restabilization thereby weakening the expression of fear 24 h later.

## Materials and Methods

### Participants

Thirty undergraduate students (8 men, 22 women) from the University of Amsterdam ranging in the age of 18–32 years (mean ± SD age, 20.9 ± 3.4 years) participated in the study. All participants were assessed to be free from any current or previous medical or psychiatric condition that would contraindicate taking a single 40 mg dose of propranolol HCl (i.e., pregnancy, seizure disorder, respiratory disorder, cardiovascular disease, blood pressure ≤ 90/60 diabetes, liver or kidney disorder, depression, psychosis). In order to eliminate individuals who might have difficulty with any temporary symptoms induced by the propranolol HCl manipulation, an additional exclusion criterion contained a score ≥ 26 on the Anxiety Sensitivity Index (ASI) (Peterson and Reiss, 1992). Participants were randomly assigned to one of two groups with the restriction that conditions were matched on Trait Anxiety (STAI-T) (Spielberger et al., 1970), Spider Phobic Questionnaire (SPQ) and Snake Phobic Questionnaire (SNAQ) (Klorman et al., 1974) as well as ASI scores as close as possible (see Table 1). Participants received either partial course credits or were paid a small amount (€35,) for their participation in the experiment. The ethical committee of the University of Amsterdam approved the study and informed consent was obtained from all participants.

**Table 1:** **Mean values (SD) of the reported spider fear (SPQ), reported snake fear (SNAQ), trait anxiety (STAI) and anxiety sensitivity (ASI) for the perceptual-learning and the categorical-learning condition**.

	Perceptual-learning	Categorical-learning
SPQ	8.2 (6.7)	9.5 (8.3)
SNAQ	6.5 (6.6)	6.9 (5.9)
STAI	34.2 (7.8)	34.7 (8.6)
ASI	8.3 (4.6)	9.3 (5.5)

### Apparatus and Materials

#### Stimuli

In order to strengthen the fear association during acquisition fear relevant stimuli served as CSs (Lang et al., 2005). Whereas we used two pictures of spiders as the CSs in the perceptual-learning group (i.e., IAPS numbers 1200–1201), two stimuli of different stimulus categories served as the CSs in the categorical-learning group (i.e., spider—snakes; IAPS numbers 1200–1050) (Lang et al., 2005). Slides were 200 mm high and 270 mm wide and were presented in the middle of a black screen on a 19-in computer monitor. Assignment of the slides as CS1 and CS2 was counterbalanced across participants. Furthermore, in the perceptual-learning group the *reactivation* stimulus (CS1-R) consisted out of the word “SPIDER”, while in the categorical-learning group the *reactivation* stimulus (CS1-R) either contained the word “SPIDER” or “SNAKE” (i.e., counterbalanced across participants). All stimuli were presented for 8 s—the startle probe was presented 7 s after CS onset and was followed by the US (CS1+) 500 ms later. An electric stimulus with duration of 2 ms delivered to the wrist of the non-preferred hand served as US. Delivery of the electric stimulus was controlled by a Digitimer DS7A constant current stimulator (Hertfordshire—UK) via a pair of Ag electrodes of 20 by 25 mm with a fixed-inter-electrode mid-distance of 45 mm. A conductive gel (Signa—Parker) was applied between the electrodes and the skin.

#### Fear Potentiated Startle Responding

Conditioned fear responding (CR) was measured as potentiation of the eyeblink startle reflex to a loud noise by electromyography (EMG) of the right orbicularis oculi muscle. Startle potentiation is considered a reliable and specific index of fear (Hamm and Weike, 2005), which is directly connected with and modulated by the amygdala (Davis, 2006). A loud noise (40 ms; 104 dB) was administered during each CS presentation and during inter-trial intervals (NA: Noise Alone). 7 mm sintered Ag-AgCl electrodes filled with electrolyte gel were positioned approximately 1 cm under the pupil and 1 cm below the lateral canthus; a ground reference was placed on the forehead (Blumenthal et al., 2005). All acoustic stimuli were delivered binaurally through headphones (Sennheiser 25 I-II). Eyeblink EMG activity was measured using a bundled pair of electrode wires connected to a front-end amplifier with an input resistance of 10 MΩ and a bandwidth of DC-1500 Hz. A notch filter was set at 50 Hz for reducing unwanted interference. Integration was handled by a true-RMS converter (contour follower) with a time constant of 25 msec. Integrated EMG signals were sampled at 1000 Hz. Peak amplitudes were identified over the period of 50–100 ms following probe onset.

#### US Expectancy Ratings

Rated expectations of the US were measured online during CS presentation using a computer mouse on a continuous rating scale placed within reach of the preferred hand. Scales consisted of 11 points labeled from “certainly no electric stimulus” (−5) through “uncertain” (0) to “certainly an electric stimulus” (5). Scores were converted into *minus* 100–100-points scales. Participants’ ratings were presented at the bottom of the computer screen in order to encourage participants to focus their attention to the CSs. Participants were required to rate the expectancy of an electric stimulus during the presentation of each slide by shifting the cursor on the scale and push the left mouse button within 5 s following stimulus onset (i.e., before presentation of the startle probe). Once the slides disappeared the cursor automatically returned to the “uncertain” position.

#### Blood Pressure

Blood pressure was measured using an electronic sphygmomanometer (OMRON M4-I) with a cuff applied around the right upper arm.

#### Pharmacological Treatment

Propranolol HCl (40 mg) pills were prepared by the pharmacy (Huygens Apotheek, Voorburg, the Netherlands).

#### Subjective Assessments

State and trait anxiety were assessed with the State and Trait Anxiety Inventory (STAI-S and STAI-T) (Spielberger et al., 1970). Spider and snake fear were determined by the SPQ and Snake Phobic Questionnaire (SNAQ) respectively (Klorman et al., 1974). The ASI (Peterson and Reiss, 1992) was used to assess one’s tendency to respond fearfully to anxiety-related symptoms.

### Experimental Procedure

Participants were subjected to a differential fear conditioning procedure including several phases across three subsequent days each separated by 24 h. During each session, participants sat behind a table with a computer monitor at a distance of 50 cm in a sound-attenuated room. Each session began with a 1 min acclimation period consisting of 70 dB broadband noise, which continued throughout the session as background noise, followed by a habituation phase consisting of ten startle probes to reduce initial startle reactivity. Characteristics of the trial order, ITIs, startle probes as well as the instructions regarding the US expectancy ratings during *memory reactivation* (i.e., day 2) and *test* (i.e., day 3) were similar to *acquisition* (i.e., day 1). Assignment of the slides as CS1 and CS2 was counterbalanced across participants. We refer to Kindt et al. (2014) for both a detailed description and visualization of our basic methodology.

#### Acquisition—Day 1

Details of the various study procedures were explained in full and possible questions were answered. The participants were interviewed regarding their health and any medical or psychiatric conditions that would contraindicate taking a single 40 mg dose of propranolol HCl. In addition, blood pressure was measured.Once a participant was medically cleared, written informed consent was obtained and the ASI, SPQ, SNAQ, and STAI were administered.

After attachment of the startle and shock electrodes the intensity of the US was determined. Starting at an intensity of 1 mA the level of a 2 ms aversive electric stimulus delivered to the wrist of the non-preferred hand was gradually increased. Intensity of shock was individually set at a level defined by the participants as “uncomfortable but not painful” and remained set to this intensity throughout the following days. After US selection participants were informed regarding the CSs. They were instructed to look carefully at both slides as an electric stimulus would follow one of the slides in most cases while the other slide would never be followed by the US. They were told to learn to predict whether an electric stimulus would occur or not on basis of the slides. Participants were required to rate the expectancy of the electric stimulus during the presentation of each slide by shifting a cursor on a continuous 11-point scale and push the left mouse button within 5 s following stimulus onset (i.e., before administration of the startle probe).

In the *acquisition* phase the CS1 and CS2 were presented 5 times for 8 s. Startle probes were presented 7 s after CS onset and were followed by the US 500 ms later (i.e., CS1+). In order to prevent that the reactivation trial on day 2 would results in extinction learning, the first presentation of the CS1 was unreinforced (LaBar et al., 1998). Furthermore, 5 baseline startle probes were presented alone (noise alone—NA) during the inter-trial intervals (ITIs). ITIs varied between 15–20–25 s with a mean of 20 s. Order of trial types and ITIs were randomized within blocks (i.e., CS1, CS2, and NA).

At the conclusion of the acquisition phase participants were asked to evaluate the pleasantness of the US and complete the STAI-S. Furthermore, they were explicitly instructed to remember what they had learned during acquisition. These instructions were included to enhance retention of the CS-US contingency on the following days (Norrholm et al., 2006) and to prevent participants from erroneously expecting a different contingency scheme during subsequent testing.

#### Memory Reactivation—Day 2

In order to substantiate consolidation of the fear memory a break of 24 h was inserted after acquisition. Subsequent to the attachment of the electrodes the participants were told that the same two slides would be presented and they were asked to remember what they had learned during acquisition. Further instructions regarding the US expectancy ratings were similar to day one. In the *memory reactivation* phase a single CS1-R was presented followed by a NA startle probe.

All of the participants received single-blind an oral dose of 40 mg of propranolol HCl *after* the reactivation of the memory. Propranolol is supposed to specifically act on the β-adrenergic receptors in the basolateral amygdala (Roozendaal, 2002; McGaugh, 2004; Johansen et al., 2011, 2014). In view of the peak plasma levels of propranolol HCl (Gilman and Goodman, 1996), a resting period of 90 min was inserted following the pill administration. Both before pill administration and upon completion of the experiment (i.e., after the resting period) participants filled out the STAI-S and blood pressure was measured.

#### Test—Day 3

Upon arriving at the experimental site, blood pressure was measured and the STAI-S was completed. Instructions regarding the CSs only revealed that the same two pictures provided during acquisition would be presented. Moreover, participants were again required to rate the expectancy of the electric stimulus during the presentation of each slide. Participants were exposed to the CS1 and CS2 as well as the NA trial only once, where after they received 3 unsignaled USs. Time between the test trials and the reinstating US was 19 s. Following the unsignaled USs participants were again presented with one CS1, CS2 and NA trial. Time between the reinstating USs and reinstatement testing was 18 s. Note that in our previous studies (e.g., Kindt et al., 2009; Sevenster et al., 2013), the reminder shocks were always presented *following* fear extinction. As such we now tested whether targeting the process of reconsolidation without fear extinction also prevented the return of fear following the reminder shocks. At the conclusion of the experiment participants completed the STAI-S and judged the pleasantness of the US.

### Statistical Analysis

Startle responses and US expectancy ratings were analyzed by means of a mixed analysis of variance (ANOVA) for repeated measures with group (i.e., perceptual-learning *vs*. categorical-learning) as between-subjects factor and stimulus (i.e., CS1* vs*. CS2) and trial (i.e., stimulus presentation) as within-subjects factors. Differential responding (CS1 *vs*. CS2) was compared over testing phases respectively (first trial *vs*. last trial). Planned comparisons were performed for each group separately. Missing startle responses (i.e., 0.4% of the trials) were excluded from the analysis. Furthermore startle responses that exceeded 2.5 standard deviations over the average peak amplitudes (i.e., 1.2% of trials) were determined as outliers and were replaced by the linear trend of that data point for each phase and CS type separately. Significance was set at *P* < 0.05.

## Results

Participants in the perceptual-learning and categorical-learning group did not differ in terms of reported spider as well as snake fear (*t*s_(28)_ < 1), trait anxiety (*t*_(28)_ < 1), anxiety sensitivity (*t*_(28)_ < 1), and shock intensity (*t*_(28)_ < 1). Selected shock intensities ranged from 6 to 35 mA with a mean of 12.5 (SD = 7.6). Furthermore, we observed no differences to the degree participants experienced the US (*t*s_(28)_ < 1).

### Manipulation Check Propranolol HCl

*Propranolol HCl* during memory reactivation did not differentially affect the systolic and diastolic BP between groups (moment × group, *F*s_(1,28)_ < 1). In both groups we observed a significant decrease in systolic (moment, *F*_(1,28)_ = 111.12, *P* < 0.001, *η*_p_^2^ = 0.80) as well as diastolic BP (moment, *F*_(1,28)_ = 52.13, *P* < 0.001, *η*_p_^2^ = 0.65) following the propranolol administration, which indicates that the drug exerted its intended physiological effect. Furthermore, BP again returned to baseline levels at retention testing (i.e., day 3) given that we observed no effect of the pill administration on the course of the systolic and diastolic BP (day 1 *vs*. day 3; moment, moment × group, *F*s_(1,28)_ < 1.63).

Consistent with other studies (Grillon et al., 2004), the propranolol HCl manipulation did not affect the reported state anxiety that was assessed before and after pill intake during memory reactivation (i.e., day 2) (moment × group, *F*s_(1,28)_ < 1). We also observed no effect of the pill administration on the reported state anxiety that was assessed before acquisition and upon arriving at the experimental site 48 h later (day 1 *vs*. day 3; moment, moment × group, *F*s_(1,28)_ < 1). We further observed no effects of the propranolol HCl manipulation on the course of the US evaluation (day 1 *vs*. day 3; moment, moment × group, *F*s_(1,28)_ < 2.75).

### US Expectancy Ratings

**Figure 2:**
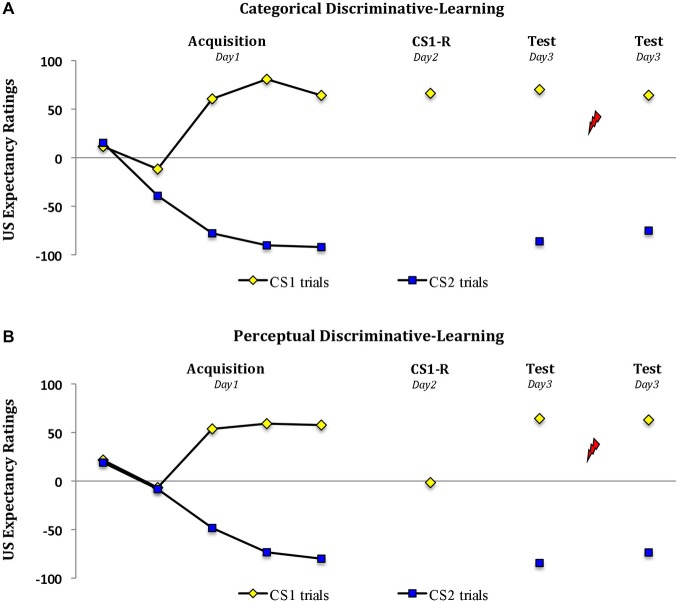
**Propranolol does not affect the US expectancy ratings**. Mean US expectancy scores to the fear-conditioned stimulus CS1 and the control stimulus CS2 trials during acquisition, memory reactivation and test for the **(A)** categorical discriminative-learning and **(B)** perceptual discriminative-learning group. A clear threat expectation during memory reactivation was observed in the **(A)** categorical discriminative-learning group. Conversely the participants in the perceptual discriminative learning group **(B)** remained uncertain about the shock probability during memory reactivation. Error bars represent SEM. Unsignaled USs are depicted as lightning bolts.

Analysis of the expectancy ratings revealed a significant increase in US expectancy during acquisition in both groups (trial 1 *vs*. trial 5; stimulus × trial, *F*_(1,28)_ = 148.22, *P* < 0.001, *η*_p_^2^ = 0.84; stimulus × trial × group, *F*_(1,28)_ < 1.06; Figure 2). However the two groups differed in their responding during memory reactivation (*t*_(28)_ = 9.48, *P* < 0.001, two-tailed). Whereas reactivation by a word cue resulted in a significant drop in US expectancy from *M* = 57.6 (SD = 25.3) during the last trial of acquisition to *M* = 1.6 (SD = 15.5) (‘uncertainty’) in the perceptual-learning group (main effect of trial, *F*_(1,14)_ = 71.44, *P* < 0.001, *η*_p_^2^ = 0.84), the threat expectancies persisted from *M* = 64.1 (SD = 29.8) at the end of acquisition to *M* = 66.3 (SD = 23.0) in the categorical-learning group (main effect of trial, *F*_(1,14)_ < 1; trial × group, *F*_(1,28)_ = 43.61, *P* < 0.001, *η*_p_^2^ = 0.61). Irrespective of this drop in threat expectation during memory reactivation in the perceptual-learning group, the two groups revealed a similar pattern of expectancies during subsequent testing 24 h later (stimulus × group, *F*s_(1,28)_ < 1). That is, in both groups we observed a differential US expectancy during the first trial at test (main effect of stimulus, *F*_(1,28)_ = 894.56, *P* < 0.001, *η*_p_^2^ = 0.97) and following the reminder shocks (main effect of stimulus, *F*_(1,28)_ = 203.63, *P* < 0.001, *η*_p_^2^ = 0.88).

### Fear Potentiated Startle Responding

**Figure 3:**
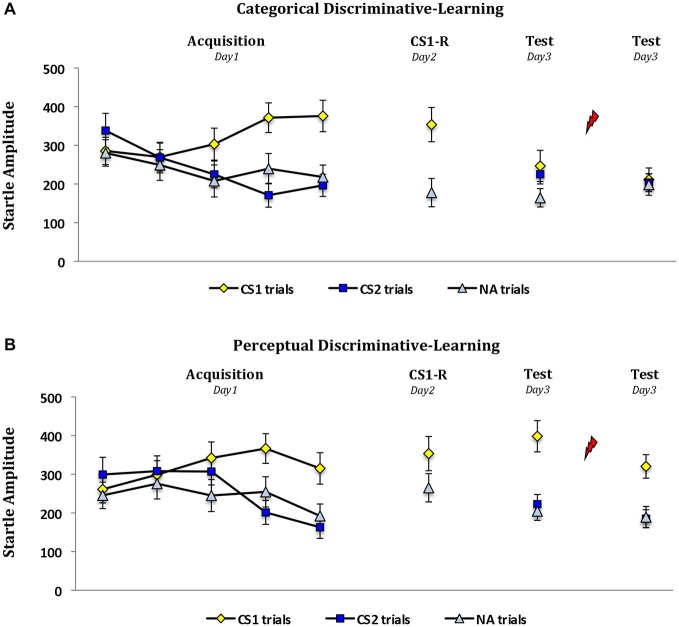
**Erasure of the startle fear responding by propranolol depends on the learning history**. Mean startle potentiation to the fear-conditioned stimulus (CS1), the control stimulus (CS2) and the noise alone (NA) trials during acquisition, memory reactivation (i.e., CS1-R) and test for the **(A)** categorical discriminative-learning and **(B)** perceptual discriminative-learning group. Propranolol affected the startle responding in **(A)** the categorical discriminative-learning, but not in **(B)** the perceptual discriminative-learning group. Error bars represent SEM. Unsignaled USs are depicted as lightning bolts.

Analysis of variance also showed similar fear learning (i.e., day 1) in the categorical and perceptual-learning group as is indicated by a significant increase of the differential startle fear responding (CS1 *vs*. CS2) from the first trial to the last trial of acquisition (stimulus × trial, *F*_(1,28)_ = 38.94, *P* < 0.001, *η*_p_^2^ = 0.58; stimulus × trial × group, *F*_(1,28)_ < 1; Figure 3). Contrary to the expectancy ratings, the two groups showed no differences in startle potentiation to the word cue during reactivation (CS1-R *vs*. NA—stimulus × group, *F*_(1,28)_ < 3.67, *P* = 0.066), though this result approached significance. But importantly this discrepancy between groups was explained by differences in responding to the NA trial presented after reactivation (*t*_(28)_ = 2.13, *P* < 0.05, two-tailed) and not to the CS1-R itself (*t*_(28)_ < 1; Figure 3). Moreover the fear expression during reactivation was significant in both groups (i.e., CS1-R *vs*. NA—perceptual-learning groups: *t*_(14)_ = 2.84, *P* < 0.05, two-tailed—categorical-learning group: *t*_(14)_ = 4.80, *P* < 0.001, two-tailed). An absence of a significant change in startle fear responding (CS1 *vs*. NA) from the last trial of acquisition to memory reactivation further demonstrates that the conditioned fear equally generalized to the abstract representation of the CS1 in both groups (stimulus × trial × group, *F*_(1,28)_ < 1). Also note that the differential startle responding remained stable from reactivation to the first trial at test in the perceptual-learning group (i.e., CS1 *vs*. NA; stimulus × trial, *F*_(1,14)_ < 2.41). However in line with our predictions the learning history critically determined the interfering effect of propranolol on memory reconsolidation (day 1 *vs*. day 3; stimulus × trial × group, *F*_(1,28)_ = 18.92, *P* < 0.001, *η*_p_^2^ = 0.40; Figure 3). Planned comparisons indeed showed that the propranolol manipulation significantly reduced the startle fear responding (CS1 *vs*. CS2) from the last acquisition trial to the first trial at test in the categorical-learning group (day 1 *vs*. day 3; stimulus × trial, *F*_(1,14)_ = 26.72, *P* < 0.001, *η*_p_^2^ = 0.66), but not in the perceptual-learning group (day 1 *vs*. day 3; stimulus × trial, *F*_(1,14)_ < 1). In the categorical-learning group the differential startle fear responding was in fact completely eliminated (day 3, *t*_(14)_ < 1.09), whereas it remained significant in the perceptual-learning group (*t*_(14)_ = 6.02, *P* < 0.001, two-tailed). Furthermore, the reminder shocks did not bring about any change in startle fear responding from the first to the second trial at test in both groups (stimulus × trial, stimulus × trial × group, *F*s_(1,27)_ < 1; Figure 3). Hence—contrary to the differential startle fear responding in the perceptual-learning group (*t*_(13)_ = 7.18, *P* < 0.001, two-tailed)—reinstatement testing did not reveal any fear responding in the categorical-learning group (*t*_(14)_ < 1.08).

## Discussion

In sum we demonstrated that an abstract representation of the feared stimulus that was not previously associated with the threat cue may trigger reconsolidation depending on the learning history. Although the two groups showed similar fear conditioning on day 1, the abstract word cue during memory reactivation on day 2 triggered only a clear threat expectation in the categorical-learning group. Conversely the participants in the perceptual-learning group remained uncertain about the shock probability during memory reactivation: this is also expressed by the higher startle fear responding to noise alone on day 2. Most pertinent to our hypothesis, the abstract word cue during reactivation left the original fear association sensitive to disruption in the categorical-learning group, which can be inferred from the diminished fear expression 24 h later. A single pill of 40 mg of propranolol administered after retrieval seemed to be highly effective in neutralizing the original CS1-US memory as the reminder shocks failed to uncover any fear responding in the categorical-learning but not the perceptual-learning group. Contrary to our previous studies on fear memory reconsolidation the reminder shocks were now presented without preceding fear extinction. Apparently the absence of a return of fear in our previous work could not be explained by any additional fear reducing effects of extinction learning (Soeter and Kindt, 2011).

A recent animal study by Dębiec et al. (2013) demonstrated that when two distinct cues are presented concomitantly during fear training, retrieval of one of the components triggers reconsolidation of the associated memory. However another study showed that a second-order conditioned cue reactivated the primary fear association (CS2 → CS1) but did not trigger reconsolidation of this core first-order conditioned fear memory (CS1 → US; Dębiec et al., 2006). Yet second-order related fear memories undergo reconsolidation when reactivated by the primary fear association (CS1 → CS2; Dębiec et al., 2006). It may therefore be suggested that for an original fear memory to undergo reconsolidation, the indirect cue should at least reactivate the representation of the anticipated disaster (i.e., US) (see also Sevenster et al., 2012). Retrieval *per se* is indeed not sufficient for destabilizing fear memory (Sevenster et al., 2012, 2013; Barreiro et al., 2013; Milton et al., 2013). Again we showed that even without a clear threat expectation the abstract retrieval cue seems to elicit a behavioral expression of fear. In fact, there is growing evidence that the mechanisms mediating the behavioral expression of fear are clearly dissociated from the mechanisms mediating the process of reconsolidation (Ben Mamou et al., 2006; Coccoz et al., 2011; Caffaro et al., 2012; Rodriguez-Ortiz et al., 2012; Sevenster et al., 2012, 2013; Balderas et al., 2013; Milton et al., 2013). Recent animal studies uncovered differential and dissociable receptors in the basolateral amygdala mediating the expression, destabilization and restabilization of previously conditioned fear memories (Barreiro et al., 2013; Milton et al., 2013). A behavioral expression of fear memory is not only dissociated from processes mediating memory reactivation (i.e., access to a memory trace), it seems also not being necessary for reconsolidation to occur (Barreiro et al., 2013). A fear expression itself during memory reactivation is thus not informative on whether the memory trace enters a labile phase.

However an obvious restriction in human fear conditioning research is that we do not have access to the mediating receptors of learning and memory. As such we are used to infer memory retrieval from the behavioral expression of fear. Without an independent index of memory destabilization, only the memory enhancing or amnesic effects of the manipulations themselves may inform us on the underlying memory processes during memory retrieval. Given that propranolol solely affects the amygdala-dependent emotional aspects of the memory and not the probability ratings of the US (e.g., Kindt et al., 2009; Soeter and Kindt, 2010, 2011), changes in US expectancy ratings may serve as an independent indicator of memory destabilization (Sevenster et al., 2013). If memory retrieval follows fully reinforced asymptotic learning episodes, changes in threat expectations from acquisition to test reflect a prediction error, which seems to be a prerequisite for reconsolidation (e.g., Lee, 2009; Díaz-Mataix et al., 2013). But when memory retrieval follows partially reinforced non-asymptotic learning episodes—as in our current study as well as several previous studies (Kindt et al., 2009; Soeter and Kindt, 2010, 2011)—a shift in threat expectancies is not necessary for post-retrieval plasticity. Hence a retrieval trial may also trigger reconsolidation without a change in expectancies after non-asymptotic learning. At least in the current fear-conditioning paradigm there should be a threat expectation at the moment of reactivation in order to trigger memory destabilization (e.g., intact CS1-US expectancies; Sevenster et al., 2012). Here we show that—subsequent to non-asymptotic CS-US contingency learning—the abstract stimulus during reactivation triggered an expectation of the US in the categorical-learning group but not in the perceptual-learning group. Apparently, as a result of the learning history, only if this abstract word triggered a specific threat expectation, the non-occurrence of the anticipated aversive event (US) could be experienced as mismatch between what was expected and what actually happened (i.e., prediction error) thereby leaving the original fear association sensitive to disruption by the propranolol HCl drug.

Depending on the available information during reactivation the disruption of memory reconsolidation may on the one hand lead to subtle modifications of fear memory. That is, disrupting reconsolidation may specifically disconnect higher-order memories from their original conditioned cues (e.g., Dębiec et al., 2006) and solely affect certain expressions of a single learned fear association such as the startle fear reflex (Kindt et al., 2009; Soeter and Kindt, 2010, 2012a,b; Sevenster et al., 2012, 2013). At the same time disrupting reconsolidation yields robust fear erasing effects that spread to secondary-related memories (Dębiec et al., 2006) as well as category-related information not directly associated with the originally feared stimulus (e.g., Soeter and Kindt, 2011, 2012a). Whether disrupting reconsolidation also radically alters vast networks of interrelated associative fear memories still remains unknown. This is particularly important considering that traumatic memories are thought to result in large and complex fear networks such that activation of one element of the network leads to activation of related elements (Foa and Kozak, 1986; Foa et al., 1989). Our present finding in humans that triggering the process of reconsolidation is *not* restricted to the original retrieval cues offers at least perspective for the application of reconsolidation in psychotherapy.

## Author Contributions

MK and MS designed the studies. MS collected and analyzed the data. MK and MS wrote the paper.

## Conflict of Interest Statement

The authors declare that the research was conducted in the absence of any commercial or financial relationships that could be construed as a potential conflict of interest.
